# In situ analysis of cross-hybridisation on microarrays and the inference of expression correlation

**DOI:** 10.1186/1471-2105-8-461

**Published:** 2007-11-26

**Authors:** Tineke Casneuf, Yves Van de Peer, Wolfgang Huber

**Affiliations:** 1 Department of Plant Systems Biology, VIB, B-9052 Ghent, Belgium; 2 Department of Molecular Genetics, Ghent University, B-9052 Ghent, Belgium; 3 EMBL – European Bioinformatics Institute, Cambridge CB10 1SD, UK

## Abstract

**Background:**

Microarray co-expression signatures are an important tool for studying gene function and relations between genes. In addition to genuine biological co-expression, correlated signals can result from technical deficiencies like hybridization of reporters with off-target transcripts. An approach that is able to distinguish these factors permits the detection of more biologically relevant co-expression signatures.

**Results:**

We demonstrate a positive relation between off-target reporter alignment strength and expression correlation in data from oligonucleotide genechips. Furthermore, we describe a method that allows the identification, from their expression data, of individual probe sets affected by off-target hybridization.

**Conclusion:**

The effects of off-target hybridization on expression correlation coefficients can be substantial, and can be alleviated by more accurate mapping between microarray reporters and the target transcriptome. We recommend attention to the mapping for any microarray analysis of gene expression patterns.

## Background

Microarrays are a valuable tool in functional genomics research. The breadth of their applications is reflected by the myriad of computational methods that have been developed for their analysis in the last decade. One popular practice is to compare expression patterns of genes by calculating correlation coefficients on expression level estimates across a set of conditions. Many downstream analysis tools are based on the presence or absence of correlation in the expression profiles of genes, like the inference of co-expression [1-5], gene regulatory [6] and Bayesian networks [7-10] and the study of gene family evolution [11,12]. From a biological point of view, these approaches are useful and informative, but here we show that if care has not been taken as to how these correlations are calculated and how the reporters for each transcript are selected, incorrect conclusions can be drawn.

A gene is represented on a microarray by one or more reporters, i. e. nucleotide sequences that are designed to uniquely match its transcript, or transcripts if different splice variants exist [13]. Affymetrix GeneChips are the most widely used microarray platform, and a wealth of data measured on these arrays is publicly available. Affymetrix reporters are 25-mer oligonucleotides whose sequence is complementary to the intended target. Each target is represented by a set of reporters, called *composite sequences *[13] or *probe set *[14]. Probe set size varies between 11 and 20, depending on the type of array, but is the same for the majority of the probe sets within one array. The signals of these different individual reporters are combined into one expression value for the probe set in a step called *summarization *[14-16].

The composition of the probe sets and the identifier of their gene transcript is contained in what is referred to as a CDF, a chip description file. Affymetrix, as array manufacturer, provides this information, and thanks to the openness of their technology specification, users can also construct their own custom-made CDFs. For Affymetrix' CDFs, probe set compositions are considered static and probe set annotation dynamic: with an updated annotation of a genome, the assignment of a probe set to a particular target gene can change, but never the content of its reporters [17]. For custom-made CDFs, this restriction is not necessary, as reporters can be arbitrarily assigned to targets.

Microarray technology confronts researchers with various challenges. Our understanding of transcriptomes is incomplete, and our estimates of which transcripts exist in a genome are constantly evolving. Therefore, for the analysis of microarray data it is important to ascertain that a reporter does in fact measure the transcript it was intended to target when the array was designed. Another concern is cross-hybridization, where transcripts other than the ones intended hybridize to a reporter. The signal that is obtained for such a reporter will be that of a combination of multiple different transcripts.

The widespread use of expression arrays encouraged different research groups to study the extent and effect of hybridization of cDNA molecules to reporters with mismatches in more detail. The cardinal importance of reporter annotation was underscored by observations made and evaluation tools developed by several research groups [18-21]. Dai et al. [21] conducted a comparative analysis of GeneChip data with original and redefined probe set definitions and described a discrepancy of 30 to 50% difference in the lists of reported genes using various analyses. These authors provide up-to-date reporter mapping files for various types of GeneChips that match individual reporters to transcripts. Based on the same observation of problematic reporter annotation, Zhang et al. [20] conducted an in-depth analysis of the reporter assignment on specific microarrays and pinpointed consistent but inaccurate signals across multiple experiments resulting from problematic reporters that are either non-specific or miss their target. They concluded that up to around 10% of the reporters on widely used arrays are non-specific in that they target multiple transcripts and another 10% miss their target.

Different efforts have also aimed to model hybridization strength and extent of cross-hybridization to improve the design of high affinity reporters that are less prone to cross-hybridization [22-25]. In addition, tools have been developed to infer the extent of cross-hybridization of individual reporter sets subsequent to data analysis [26].

The technical aspect of the microarray technology has also been tackled: Eklund et al. [27] reported that replacing cRNA with cDNA hybridization targets substantially reduces cross-hybridization. Alternative technologies to detect cross-hybridization on microarrays have also been suggested [28].

Wren et al. [29] described a positive relationship between the observed signal and the amount of contiguous hydrogen bonds involved in duplex formation during reporter-transcript binding. Okoniewski and Miller [30] conducted a large-scale analysis to map all interactions between reporters, probe sets and transcripts on the HGUI33A array. First, a set of basic motifs were defined to identify families of interacting probe sets as in some cases a reporter can bind more than one transcript, or a transcript can bind more than one reporter. The motifs were then used to build a bipartite graph of interactions with the probe sets and transcripts as nodes and matches as edges. The authors were able to identify several hub probe sets, whose expression combines the signals of many available transcripts. A detailed investigation of the expression signals revealed that reporters targeting multiple transcripts had higher absolute expression signal than those targeting a unique transcript, and that probe sets that contain reporters with multiple matches had increased expression correlation between them.

A different approach *in situ *was taken by Wu et al. [23] for the construction of a free energy model for cross-hybridization. These authors observed a clear relationship between the known concentrations of spiked-in transcripts in different experiments and the measured signals of reporters not designed to target these specific transcripts. Based on the sequences of these affected reporters, the authors constructed a free energy model to assess the sequence dependence of cross-hybridization which can be used to refine the algorithms used in reporter design.

These different studies intelligibly show that cross-hybridization is a critical concern for microarray analysis. It is clear that a reporter can bind different transcripts or that a transcript can bind to different reporters if stable, partial binding occurs or if hairpin structures are formed [31]. As a result, the signals of the reporters a transcript binds will be similar and correlation coefficients, calculated on these signals during downstream analysis, will be artifactual. The *in situ *effect of sequence similarity on expression correlation is however not known.

For this study we worked with the ATH1 Affymetrix GeneChip that was designed for the analysis of gene expression in *Arabidopsis thaliana*. *Arabidopsis *is the most commonly studied model plant organism and a wealth of high quality data has been generated with this GeneChip. We investigated the relationship between reporter-to-transcript sequence similarity and correlation of expression signals. We assessed the extent to which inclusion of off-target reporters in probe sets, i. e. reporters that are highly alignable to another transcript than the intended one, influences this correlation. The conventional probe set design, as defined by the manufacturer of the microarray was evaluated with respect to cross-hybridization and compared to our custom-made probe set composition.

We show that numerous probe sets on a widely used commercial array contain off-target reporters, and that inclusion of these reporters in a probe set gives rise to a signal pattern that is highly similar to that of the unintended probe set. We illustrate our findings with examples and demonstrate the effect of individual reporters through simulation. Furthermore, we put forward a novel method to detect unreliable probe set to transcript hybridization events. Our results show that excluding reporters that align well to another transcript diminishes this effect to a substantial extent and provides a method to pinpoint the occurrence of cross-hybridization in existing microarray datasets. We conclude from this study that reporter-to-transcript sequence alignment strength can be a source of error in studies of correlation of expression signals and that proper probe set composition is effective in minimizing the effect of cross-hybridization.

## Results and Discussion

### Two definitions of probe set annotation

The ATH1 is an Affymetrix GeneChip for the analysis of gene expression in the premier plant model organism *Arabidopsis thaliana*. A wealth of high quality data measured with this array is publicly available and has been widely used for various applications, such as the inference of gene co-expression networks and the study of functional aspects of the evolution of gene families [1-5,11,12] (reviewed in [32]).

For the Affymetrix CDF of the ATH1, a probe set was assigned to a gene if nine or more of its reporters had perfect sequence identity with the gene's transcript consensus sequence. If this condition was fulfilled for multiple genes, the probe set was assigned to all of them. In this way, 22,810 probe sets were assigned to more than 24,000 genes. A probe set can thus contain up to eight reporters that align perfectly to another gene's transcript without being assigned to it [17].

We built a custom-made CDF with alternative probe set definitions and annotations. We aligned each 25-mer reporter sequence to the predicted transcripts of *Arabidopsis thaliana *(see Methods for details). A reporter was assigned to a gene if it had perfect sequence identity with its transcript(s) and did not align to any other gene's transcript with zero or one mismatches. We removed reporters that had multiple hits in the genome, and reporters that had hits in the reverse complementary direction. Probe sets were defined as eight or more reporters all assigned to a particular gene's transcript(s). This resulted in 19,937 probe sets with unique assignments to 19,937 target genes. Table 1 shows some statistics on the probe set definitions. The approach we took is highly similar to the one introduced by Dai et al. [21].

**Table 1:** Statistics of probe set definitions. The first 2 rows contain the number of probe sets and reporters in the Affymetrix and the custom-made CDF. The number of reporters times the number of predicted transcripts, in the bottom row, results in the total number of reporter-to-transcript alignment scores (see also Figure 1).

	CDF Affymetrix	Custom-made CDF
Number of probe sets:	22,810	19,937
Number of reporters:	251,078	217,811
Number of alignment scores:	6,926,739,864	6,008,969,868

Total number of transcripts in TAIR6:	27,588	

In those cases where their probe set annotations are based on the UniGene database, Dai and colleagues require perfect hits to unigene clusters and unique hits of a reporter to a genomic location. For their CDFs that are based on databases other than UniGene, the rule of one transcript assignment per reporter does not apply [21], so reporters can be assigned to multiple transcripts. As this is currently the case for the ATH1 array, for which the CDF of Dai et al. is based on the TAIR annotation, we computed a custom CDF that requires uniqueness. Hence, we expect that our results can be generalized to other arrays for which Dai et al. have computed CDFs with 1:1 reporter-target mapping, and in the future, when their ATH1 CDF will be changed to unique 1:1 mapping (personal communication), it could be used instead of our custom CDF.

### Off-target alignments

**Figure 1:**
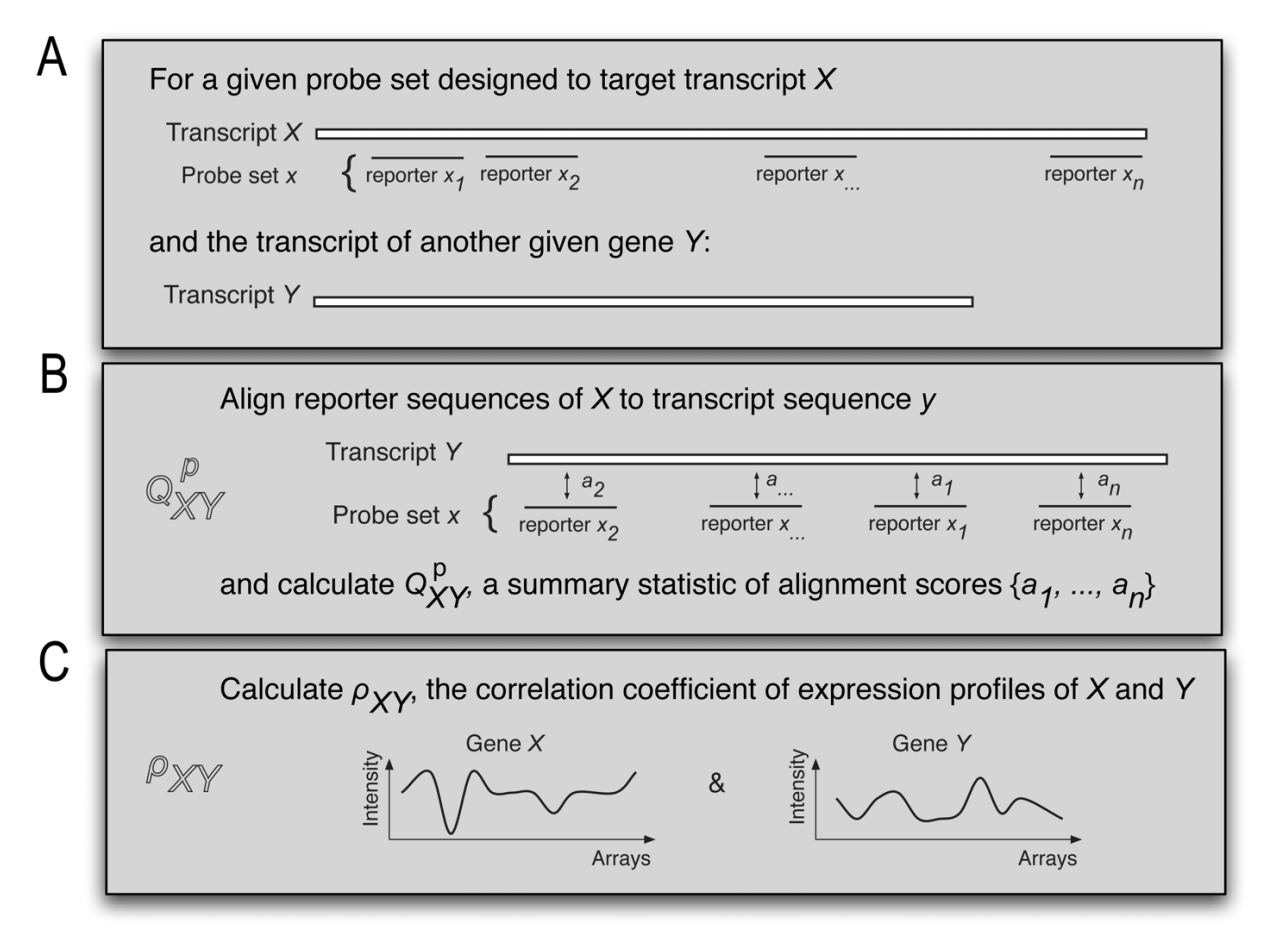
**Setup of our study**. Illustration of our approach: A) for a given probe set *x*, assigned to measure the expression of gene *X *and the transcript of a given gene *Y*, two variables QXYp
 MathType@MTEF@5@5@+=feaafiart1ev1aaatCvAUfKttLearuWrP9MDH5MBPbIqV92AaeXatLxBI9gBaebbnrfifHhDYfgasaacPC6xNi=xH8viVGI8Gi=hEeeu0xXdbba9frFj0xb9qqpG0dXdb9aspeI8k8fiI+fsY=rqGqVepae9pg0db9vqaiVgFr0xfr=xfr=xc9adbaqaaeGacaGaaiaabeqaaeqabiWaaaGcbaGaemyuae1aa0baaSqaaiabdIfayjabdMfazbqaaiabdchaWbaaaaa@3108@ and *ρ*_*XY *_were calculated. B) QXYp
 MathType@MTEF@5@5@+=feaafiart1ev1aaatCvAUfKttLearuWrP9MDH5MBPbIqV92AaeXatLxBI9gBaebbnrfifHhDYfgasaacPC6xNi=xH8viVGI8Gi=hEeeu0xXdbba9frFj0xb9qqpG0dXdb9aspeI8k8fiI+fsY=rqGqVepae9pg0db9vqaiVgFr0xfr=xfr=xc9adbaqaaeGacaGaaiaabeqaaeqabiWaaaGcbaGaemyuae1aa0baaSqaaiabdIfayjabdMfazbqaaiabdchaWbaaaaa@3108@ is a summary statistic (e. g. *p *= 75 for the 75% percentile) of the alignment scores of the reporters of *X *to the transcript of *Y*. C) *ρ*_*XY *_is the correlation coefficient of the expression signals of genes *X *and *Y*. This procedure was repeated for each probe set against every other transcript of the *Arabidopsis *transcriptome.

Our aim was to investigate the relationship between correlation coefficients of microarray gene expression profiles and potential off-target sensitivity of reporters and probe sets. Figures 1A and 1B explain our procedure of calculating the score for off-target sensitivity. For a probe set with *n *reporters designed to target gene *X*, and another gene *Y*, we computed the alignment scores {*a*_1_,...,*a*_*n*_} of *X*'s reporters to *Y*'s transcript sequence(s) with *Needle *[33], a Needleman-Wunsch alignment [34] program. A global alignment algorithm was used to align the full length of the reporter to the target while allowing for gaps and hairpin-forming. Furthermore, we used an exact algorithm to ensure that the optimal alignment was reached. *Needle *scores an identical match with a positive score of 5 and penalizes a mismatch score with *-*4. The gap open penalty was set to -50 and gap extension penalty to -0.5. The reporters have a length of 25, so a perfectly matching reporter will have a score of 125. Some interesting scores are shown in Table 2.

**Table 2:** Table with some of the highest Needleman-Wunsch scores. P and M stand for the number of perfect and mismatch scores. Gap openings and extensions in the alignment were penalized with -50 and -0.5, respectively.

Matches			Matches			Matches			Matches		
P	M	Score	P	M	Score	P	M	Score	P	M	Score

25	0	125	22	2	102	19	1	91	18	2	82
24	0	120	21	1	101	18	0	90	17	1	81
24	1	116	20	0	100	21	4	89	20	5	80
23	0	115	22	3	98	20	3	88	16	0	80
23	1	111	21	2	97	19	2	87	19	4	79
22	0	110	20	1	96	18	1	86	18	3	78
23	2	107	19	0	95	17	0	85	17	2	77
22	1	106	21	3	93	20	4	84	16	1	76
21	0	105	20	2	92	19	3	83	19	5	75

To quantify the potential off-target affinity of a probe set, different percentiles QXYp
 MathType@MTEF@5@5@+=feaafiart1ev1aaatCvAUfKttLearuWrP9MDH5MBPbIqV92AaeXatLxBI9gBaebbnrfifHhDYfgasaacPC6xNi=xH8viVGI8Gi=hEeeu0xXdbba9frFj0xb9qqpG0dXdb9aspeI8k8fiI+fsY=rqGqVepae9pg0db9vqaiVgFr0xfr=xfr=xc9adbaqaaeGacaGaaiaabeqaaeqabiWaaaGcbaGaemyuae1aa0baaSqaaiabdIfayjabdMfazbqaaiabdchaWbaaaaa@3108@ were calculated of the reporter alignment scores {*a*_1_,...,*a*_*n*_}, where *p *∈ [0, 100] is the percentile, *X *is the intended target gene of the probe set and *Y *is the potential off-target. For the results presented in this paper, we used *p *= 75, but qualitatively equivalent results were obtained with other values of *p*.

This analysis was carried out for each probe set against every sequence of the transcriptome of *Arabidopsis *(as found in the TAIR6 sequence database [35]), which results in a total number of 6,926,739,864 alignments for the Affymetrix CDF and 6,008,969,868 for the custom-made CDF (see Table 1). Additional File 1 shows a histogram of the highest alignment scores of the pairs of the two CDFs.

### Correlation of microarray expression profiles

Pearson correlation coefficients, *ρ*_*XY *_were calculated for every pair of probe sets *X *and *Y *on two different ATH1 microarray datasets. One dataset contains expression data in 14 different plant tissues and the other is a dataset of nine stress conditions and consists of 60 datapoints (see Methods). Both datasets were generated by the AtGenExpress project [36].

### Probe set off-target sensitivity and expression correlation

**Figure 2:**
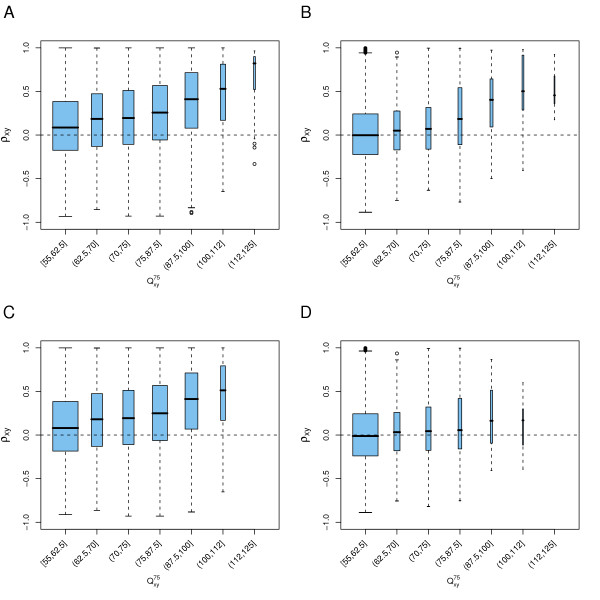
**Probe set off-target sensitivity and expression correlation**. Boxplots depicting the expression correlation coefficients, *ρ*_*XY *_stratified by off-target sensitivity score, QXY75
 MathType@MTEF@5@5@+=feaafiart1ev1aaatCvAUfKttLearuWrP9MDH5MBPbIqV92AaeXatLxBI9gBaebbnrfifHhDYfgasaacPC6xNi=xH8viVGI8Gi=hEeeu0xXdbba9frFj0xb9qqpG0dXdb9aspeI8k8fiI+fsY=rqGqVepae9pg0db9vqaiVgFr0xfr=xfr=xc9adbaqaaeGacaGaaiaabeqaaeqabiWaaaGcbaGaemyuae1aa0baaSqaaiabdIfayjabdMfazbqaaiabiEda3iabiwda1aaaaaa@3193@. Figures A and C show the data for all probe set pairs; for Figures B and D gene pairs with a BLAST hit in at least one direction with an E-value smaller than 10^-10 ^were omitted. A-B) Results obtained with Affymetrix' CDF. C-D) Results obtained with the custom-made CDF. The widths of the boxes are proportional to the number of observations in each group. *ρ*_*XY *_was calculated on the tissue microarray dataset. The plots show results for all pairs with QXY75
 MathType@MTEF@5@5@+=feaafiart1ev1aaatCvAUfKttLearuWrP9MDH5MBPbIqV92AaeXatLxBI9gBaebbnrfifHhDYfgasaacPC6xNi=xH8viVGI8Gi=hEeeu0xXdbba9frFj0xb9qqpG0dXdb9aspeI8k8fiI+fsY=rqGqVepae9pg0db9vqaiVgFr0xfr=xfr=xc9adbaqaaeGacaGaaiaabeqaaeqabiWaaaGcbaGaemyuae1aa0baaSqaaiabdIfayjabdMfazbqaaiabiEda3iabiwda1aaaaaa@3193@ ≥ 55.

The relation between expression correlation, *ρ*_*XY *_and off-target sensitivity, QXY75
 MathType@MTEF@5@5@+=feaafiart1ev1aaatCvAUfKttLearuWrP9MDH5MBPbIqV92AaeXatLxBI9gBaebbnrfifHhDYfgasaacPC6xNi=xH8viVGI8Gi=hEeeu0xXdbba9frFj0xb9qqpG0dXdb9aspeI8k8fiI+fsY=rqGqVepae9pg0db9vqaiVgFr0xfr=xfr=xc9adbaqaaeGacaGaaiaabeqaaeqabiWaaaGcbaGaemyuae1aa0baaSqaaiabdIfayjabdMfazbqaaiabiEda3iabiwda1aaaaaa@3193@ is shown in Figure 2. Figure 2A shows the results we obtained with all probe set pairs of the Affymetrix CDF and Figure 2C shows those of the custom-made CDF. These boxplots reveal a positive relation between the two variables: a gene whose expression is measured by reporters that align well to a different gene's transcript tends to have an expression signal that is correlated with that of the other gene.

Because a positive trend between (reporter) alignment strength and expression correlation is not unexpected for functionally related genes like paralogous genes or genes that share protein domains, we defined a filtering criterion to set aside gene pairs that aligned to each other with BLAST [37] in at least one direction with an E-value smaller than 10^-10 ^(see Methods). Figure 2B and Figure 2D show the data for the remaining probe set pairs of the Affymetrix and the custom-made CDF, respectively. For both, we see that for QXY75
 MathType@MTEF@5@5@+=feaafiart1ev1aaatCvAUfKttLearuWrP9MDH5MBPbIqV92AaeXatLxBI9gBaebbnrfifHhDYfgasaacPC6xNi=xH8viVGI8Gi=hEeeu0xXdbba9frFj0xb9qqpG0dXdb9aspeI8k8fiI+fsY=rqGqVepae9pg0db9vqaiVgFr0xfr=xfr=xc9adbaqaaeGacaGaaiaabeqaaeqabiWaaaGcbaGaemyuae1aa0baaSqaaiabdIfayjabdMfazbqaaiabiEda3iabiwda1aaaaaa@3193@ values of up to around 70, the distribution of signal correlations of the probe set pairs is centered around zero. Pairs with higher QXY75
 MathType@MTEF@5@5@+=feaafiart1ev1aaatCvAUfKttLearuWrP9MDH5MBPbIqV92AaeXatLxBI9gBaebbnrfifHhDYfgasaacPC6xNi=xH8viVGI8Gi=hEeeu0xXdbba9frFj0xb9qqpG0dXdb9aspeI8k8fiI+fsY=rqGqVepae9pg0db9vqaiVgFr0xfr=xfr=xc9adbaqaaeGacaGaaiaabeqaaeqabiWaaaGcbaGaemyuae1aa0baaSqaaiabdIfayjabdMfazbqaaiabiEda3iabiwda1aaaaaa@3193@ values are however accompanied by elevated signal correlation, even though for the gene pairs no functional relation is suggested by their sequence comparison. For a probe set with 11 reporters, the QXY75
 MathType@MTEF@5@5@+=feaafiart1ev1aaatCvAUfKttLearuWrP9MDH5MBPbIqV92AaeXatLxBI9gBaebbnrfifHhDYfgasaacPC6xNi=xH8viVGI8Gi=hEeeu0xXdbba9frFj0xb9qqpG0dXdb9aspeI8k8fiI+fsY=rqGqVepae9pg0db9vqaiVgFr0xfr=xfr=xc9adbaqaaeGacaGaaiaabeqaaeqabiWaaaGcbaGaemyuae1aa0baaSqaaiabdIfayjabdMfazbqaaiabiEda3iabiwda1aaaaaa@3193@ summary statistic with *p *= 75 corresponds to the third strongest off-target reporter. A reporter alignment score value larger than 70 results from 15 or more perfect matches (cf. Table 2). Hence, our results imply that three or more well-aligning off-target reporters in a probe set are associated with elevated expression correlation. Figures 2A and 2B also reveal that some probe sets in the Affymetrix CDF contain three or more reporters with perfect sequence identity to an off-target gene. These probe sets are in the rightmost boxes of these figures, corresponding to the score interval (112, 125]. The custom-made CDF does not contain such reporters, since all reporters uniquely map to their target gene's transcript and have at least two mismatches with any other sequence. As a result, the rightmost score interval in Figures 2C and 2D does not contain any probe sets, and the second-highest interval (100, 112] contains only a few. A slight trend however remains. The results shown in Figure 2 were calculated on the tissue dataset, similar results were obtained for the stress dataset. Different forces can give rise to the trend we observe here. First of all, genes with partially similar sequences can show biologically relevant expression correlation. Even though many such pairs will have been removed by the above filtering criterion, some may still remain in our dataset. Second, the trend can be due to cross-hybridization, where the cDNA of a gene's transcript binds to both the reporters of its own probe set and those of other genes' probe sets. Both effects, functional relatedness and cross-hybridization, can play at the same time.

### Reporter off-target sensitivity and expression correlation

In an attempt to discern cross-hybridization from functional relatedness and to identify incidences of unreliable reporter to transcript hybridization, we designed a method that studies the behavior of off-target sensitivity and signal correlation of different reporters within a probe set. For a probe set *X *and an off-target gene *Y*, we calculated the metacorrelation cor(ρXiY
 MathType@MTEF@5@5@+=feaafiart1ev1aaatCvAUfKttLearuWrP9MDH5MBPbIqV92AaeXatLxBI9gBaebbnrfifHhDYfgasaacPC6xNi=xH8viVGI8Gi=hEeeu0xXdbba9frFj0xb9qqpG0dXdb9aspeI8k8fiI+fsY=rqGqVepae9pg0db9vqaiVgFr0xfr=xfr=xc9adbaqaaeGacaGaaiaabeqaaeqabiWaaaGcbaacciGae8xWdi3aaSbaaSqaaiabdIfaynaaBaaameaacqWGPbqAaeqaaSGaemywaKfabeaaaaa@31CD@, *a*_*i*_) between the alignment scores *a*_*i *_of *X*'s reporters to *Y*'s transcript sequence and the Pearson correlation coefficients of the reporters' signal patterns to the expression pattern of *Y*. We reasoned that if cross-hybridization occurs, a positive trend between reporter to off-target correlation and the alignment score *a*_*i *_can be detected. Conversely, lack of such a trend may indicate that cross-hybridization is negligible.

**Figure 3:**
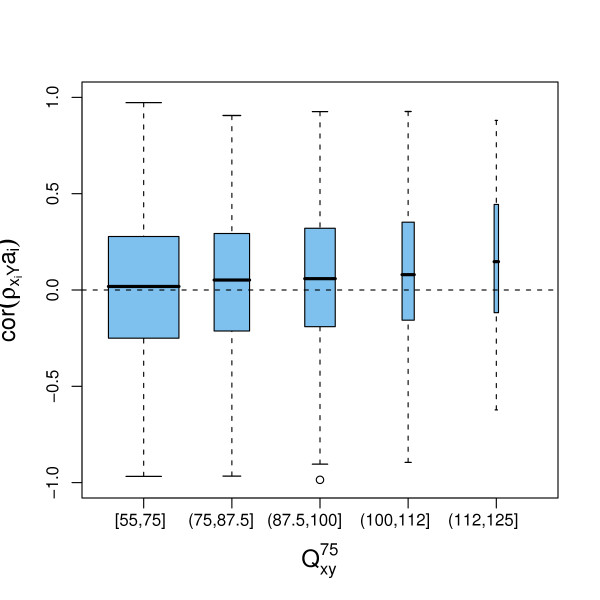
**Reporter off-target sensitivity and expression correlation**. A boxplot showing the metacorrelation coefficients cor(ρXiY
 MathType@MTEF@5@5@+=feaafiart1ev1aaatCvAUfKttLearuWrP9MDH5MBPbIqV92AaeXatLxBI9gBaebbnrfifHhDYfgasaacPC6xNi=xH8viVGI8Gi=hEeeu0xXdbba9frFj0xb9qqpG0dXdb9aspeI8k8fiI+fsY=rqGqVepae9pg0db9vqaiVgFr0xfr=xfr=xc9adbaqaaeGacaGaaiaabeqaaeqabiWaaaGcbaacciGae8xWdi3aaSbaaSqaaiabdIfaynaaBaaameaacqWGPbqAaeqaaSGaemywaKfabeaaaaa@31CD@, *a*_*i*_) of all probe set pairs of the Affymetrix CDF, stratified by their off-target sensitivity score QXY75
 MathType@MTEF@5@5@+=feaafiart1ev1aaatCvAUfKttLearuWrP9MDH5MBPbIqV92AaeXatLxBI9gBaebbnrfifHhDYfgasaacPC6xNi=xH8viVGI8Gi=hEeeu0xXdbba9frFj0xb9qqpG0dXdb9aspeI8k8fiI+fsY=rqGqVepae9pg0db9vqaiVgFr0xfr=xfr=xc9adbaqaaeGacaGaaiaabeqaaeqabiWaaaGcbaGaemyuae1aa0baaSqaaiabdIfayjabdMfazbqaaiabiEda3iabiwda1aaaaaa@3193@. Only pairs with QXY75
 MathType@MTEF@5@5@+=feaafiart1ev1aaatCvAUfKttLearuWrP9MDH5MBPbIqV92AaeXatLxBI9gBaebbnrfifHhDYfgasaacPC6xNi=xH8viVGI8Gi=hEeeu0xXdbba9frFj0xb9qqpG0dXdb9aspeI8k8fiI+fsY=rqGqVepae9pg0db9vqaiVgFr0xfr=xfr=xc9adbaqaaeGacaGaaiaabeqaaeqabiWaaaGcbaGaemyuae1aa0baaSqaaiabdIfayjabdMfazbqaaiabiEda3iabiwda1aaaaaa@3193@ ≥ 55 are included. The correlation coefficients were calculated on the intensities measured in the tissue dataset.

Figure 3 depicts this metacorrelation coefficient for all probe set pairs with QXY75
 MathType@MTEF@5@5@+=feaafiart1ev1aaatCvAUfKttLearuWrP9MDH5MBPbIqV92AaeXatLxBI9gBaebbnrfifHhDYfgasaacPC6xNi=xH8viVGI8Gi=hEeeu0xXdbba9frFj0xb9qqpG0dXdb9aspeI8k8fiI+fsY=rqGqVepae9pg0db9vqaiVgFr0xfr=xfr=xc9adbaqaaeGacaGaaiaabeqaaeqabiWaaaGcbaGaemyuae1aa0baaSqaaiabdIfayjabdMfazbqaaiabiEda3iabiwda1aaaaaa@3193@ ≥ 55 of the Affymetrix CDF stratified by their off-target sensitivity score QXY75
 MathType@MTEF@5@5@+=feaafiart1ev1aaatCvAUfKttLearuWrP9MDH5MBPbIqV92AaeXatLxBI9gBaebbnrfifHhDYfgasaacPC6xNi=xH8viVGI8Gi=hEeeu0xXdbba9frFj0xb9qqpG0dXdb9aspeI8k8fiI+fsY=rqGqVepae9pg0db9vqaiVgFr0xfr=xfr=xc9adbaqaaeGacaGaaiaabeqaaeqabiWaaaGcbaGaemyuae1aa0baaSqaaiabdIfayjabdMfazbqaaiabiEda3iabiwda1aaaaaa@3193@. The results for the custom-made CDF are similar, except for the highest score interval (112, 125], which does not occur with the custom-made CDF. The distribution of the metacorrelations of most probe set pairs corresponds to a random distribution centered around zero. However, for those strata with high off-target sensitivity scores the distribution is shifted upwards. This means that within these probe sets some reporters do not correlate with the off-target, while others do, depending on their alignments score.

### Examples

**Figure 4:**
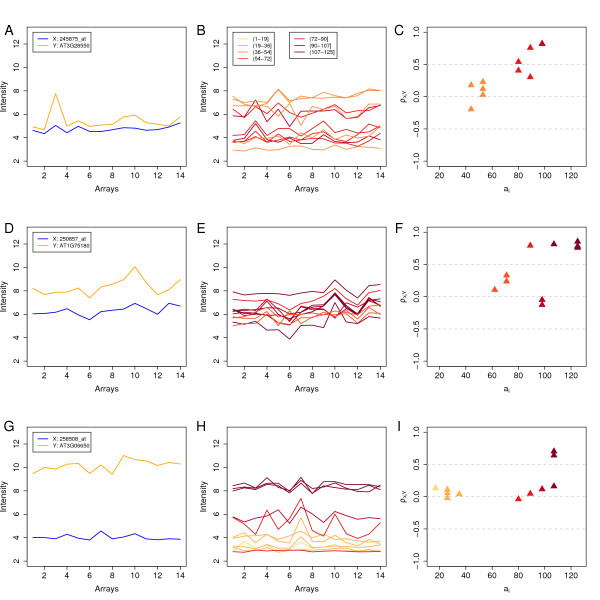
**Examples**. Each of the three rows presents an example of cross-hybridization. Each time, the first of the plots (A-D-G) shows the summarized expression values of probe set *X *(in blue) and probe set *Y *(in orange) in 14 different plant tissues. The plots in the second column (B-E-H) present the background corrected, normalized expression patterns of *X*'s reporters. The signal profile of the reporter is plotted in a color that corresponds to its alignment score to *Y *and is explained in the legend of plot B. In the third column (C-F-I) for each of *X*'s reporters, ρXiY
 MathType@MTEF@5@5@+=feaafiart1ev1aaatCvAUfKttLearuWrP9MDH5MBPbIqV92AaeXatLxBI9gBaebbnrfifHhDYfgasaacPC6xNi=xH8viVGI8Gi=hEeeu0xXdbba9frFj0xb9qqpG0dXdb9aspeI8k8fiI+fsY=rqGqVepae9pg0db9vqaiVgFr0xfr=xfr=xc9adbaqaaeGacaGaaiaabeqaaeqabiWaaaGcbaacciGae8xWdi3aaSbaaSqaaiabdIfaynaaBaaameaacqWGPbqAaeqaaSGaemywaKfabeaaaaa@31CD@, calculated between its signal profile to that of *Y*, is plotted against its alignment score, aXiY
 MathType@MTEF@5@5@+=feaafiart1ev1aaatCvAUfKttLearuWrP9MDH5MBPbIqV92AaeXatLxBI9gBaebbnrfifHhDYfgasaacPC6xNi=xH8viVGI8Gi=hEeeu0xXdbba9frFj0xb9qqpG0dXdb9aspeI8k8fiI+fsY=rqGqVepae9pg0db9vqaiVgFr0xfr=xfr=xc9adbaqaaeGacaGaaiaabeqaaeqabiWaaaGcbaGaemyyae2aaSbaaSqaaiabdIfaynaaBaaameaacqWGPbqAaeqaaSGaemywaKfabeaaaaa@3151@. Colors correspond to those used in the plot in the second column.

The metacorrelation method we developed was used to search for examples that illustrate our findings. Three examples are discussed in detail, each of which are presented in a row of Figure 4. The plots in the first column of this figure contain the summarized expression values of a probe set *X *(in blue) and an off-target gene *Y *(in orange) in the tissue dataset. The plots in the second column show the background corrected, normalized signal profiles of *X*'s reporters. The color used to plot such a profile corresponds to the alignment score of that reporter to *Y*'s transcript and is explained in the legend in Figure 4B. In the third column, for each reporter ρXiY
 MathType@MTEF@5@5@+=feaafiart1ev1aaatCvAUfKttLearuWrP9MDH5MBPbIqV92AaeXatLxBI9gBaebbnrfifHhDYfgasaacPC6xNi=xH8viVGI8Gi=hEeeu0xXdbba9frFj0xb9qqpG0dXdb9aspeI8k8fiI+fsY=rqGqVepae9pg0db9vqaiVgFr0xfr=xfr=xc9adbaqaaeGacaGaaiaabeqaaeqabiWaaaGcbaacciGae8xWdi3aaSbaaSqaaiabdIfaynaaBaaameaacqWGPbqAaeqaaSGaemywaKfabeaaaaa@31CD@, the Pearson correlation coefficient calculated between its signal profile and that of *Y *(orange in A-D-G) is plotted in function of its alignment score aXiY
 MathType@MTEF@5@5@+=feaafiart1ev1aaatCvAUfKttLearuWrP9MDH5MBPbIqV92AaeXatLxBI9gBaebbnrfifHhDYfgasaacPC6xNi=xH8viVGI8Gi=hEeeu0xXdbba9frFj0xb9qqpG0dXdb9aspeI8k8fiI+fsY=rqGqVepae9pg0db9vqaiVgFr0xfr=xfr=xc9adbaqaaeGacaGaaiaabeqaaeqabiWaaaGcbaGaemyyae2aaSbaaSqaaiabdIfaynaaBaaameaacqWGPbqAaeqaaSGaemywaKfabeaaaaa@3151@. The colors are identical to those used in the second column.

Probe set *X *in our first example is *245875_at*, which was designed to target gene *AT1G26240*, an extensin-like family protein. As shown in Figure 4A, the expression profile of this gene resembles that of *AT3G28550*, a protein that belongs to a zinc finger family. The Pearson correlation coefficient of these expression patterns is 0.63 in the tissue and 0.62 in the stress dataset. Figures 4B and 4C show that six of *X*'s reporters with aXiY
 MathType@MTEF@5@5@+=feaafiart1ev1aaatCvAUfKttLearuWrP9MDH5MBPbIqV92AaeXatLxBI9gBaebbnrfifHhDYfgasaacPC6xNi=xH8viVGI8Gi=hEeeu0xXdbba9frFj0xb9qqpG0dXdb9aspeI8k8fiI+fsY=rqGqVepae9pg0db9vqaiVgFr0xfr=xfr=xc9adbaqaaeGacaGaaiaabeqaaeqabiWaaaGcbaGaemyyae2aaSbaaSqaaiabdIfaynaaBaaameaacqWGPbqAaeqaaSGaemywaKfabeaaaaa@3151@ ≥ 80 have a signal profile that is highly correlated with that of *AT3G28550*. The remaining five have lower off-target sensitivity values and have a signal profile that is correlated less well with it. The QXY75
 MathType@MTEF@5@5@+=feaafiart1ev1aaatCvAUfKttLearuWrP9MDH5MBPbIqV92AaeXatLxBI9gBaebbnrfifHhDYfgasaacPC6xNi=xH8viVGI8Gi=hEeeu0xXdbba9frFj0xb9qqpG0dXdb9aspeI8k8fiI+fsY=rqGqVepae9pg0db9vqaiVgFr0xfr=xfr=xc9adbaqaaeGacaGaaiaabeqaaeqabiWaaaGcbaGaemyuae1aa0baaSqaaiabdIfayjabdMfazbqaaiabiEda3iabiwda1aaaaaa@3193@ value of *245875_at *to *AT3G28550 *is 89, the metacorrelation coefficient of the reporters of *245875_at *is 0.89.

The second example is of probe set *250857_at*, which was designed for *AT5G04790*, and gene *AT1G75180*. The function of both genes is unknown. Their *ρ*_*XY *_is 0.70 and 0.89 in the tissue (in Figure 4D) and stress dataset respectively. Figures 4E and 4F reveal a positive relationship between off-target sensitivity and signal correlation. Interestingly, four reporters of probe set *250857_at *have 25 identical matches to *AT1G75180 *and show an expression profile with *ρ *> 0.8. Two other reporters, with lower sensitivity to this off-target (107 and 89) also show high signal correlation to it. The QXY75
 MathType@MTEF@5@5@+=feaafiart1ev1aaatCvAUfKttLearuWrP9MDH5MBPbIqV92AaeXatLxBI9gBaebbnrfifHhDYfgasaacPC6xNi=xH8viVGI8Gi=hEeeu0xXdbba9frFj0xb9qqpG0dXdb9aspeI8k8fiI+fsY=rqGqVepae9pg0db9vqaiVgFr0xfr=xfr=xc9adbaqaaeGacaGaaiaabeqaaeqabiWaaaGcbaGaemyuae1aa0baaSqaaiabdIfayjabdMfazbqaaiabiEda3iabiwda1aaaaaa@3193@ value of probe set *250857_at *to gene *AT1G75180 *is 125, the metacorrelation coefficient of the reporters of *250857_at *is 0.62.

Figure 4G shows the expression patterns of probe set *258508_at *and *AT3G06650*. *258508_at *was designed to target *AT3G06640*, a protein kinase family protein. *AT3G06650 *is a gene that encodes a subunit of the trimeric enzyme ATP citrate lyase. *AT3G06650 *and *AT3G06640 *are neighboring genes that align for a stretch of about 50 base pairs with sequence similarity of >90%. The Pearson correlation coefficients of their expression profiles in the tissue and stress dataset are 0.30 and 0.16, respectively. Three reporters of *258508_at *have an off-target sensitivity to *AT3G06650 *of 107 (Figure 4H and 4I). Two of them have a ρXiY
 MathType@MTEF@5@5@+=feaafiart1ev1aaatCvAUfKttLearuWrP9MDH5MBPbIqV92AaeXatLxBI9gBaebbnrfifHhDYfgasaacPC6xNi=xH8viVGI8Gi=hEeeu0xXdbba9frFj0xb9qqpG0dXdb9aspeI8k8fiI+fsY=rqGqVepae9pg0db9vqaiVgFr0xfr=xfr=xc9adbaqaaeGacaGaaiaabeqaaeqabiWaaaGcbaacciGae8xWdi3aaSbaaSqaaiabdIfaynaaBaaameaacqWGPbqAaeqaaSGaemywaKfabeaaaaa@31CD@ ≥ 0.6, but the mean intensity of all three is higher than that of the other reporters. The QXY75
 MathType@MTEF@5@5@+=feaafiart1ev1aaatCvAUfKttLearuWrP9MDH5MBPbIqV92AaeXatLxBI9gBaebbnrfifHhDYfgasaacPC6xNi=xH8viVGI8Gi=hEeeu0xXdbba9frFj0xb9qqpG0dXdb9aspeI8k8fiI+fsY=rqGqVepae9pg0db9vqaiVgFr0xfr=xfr=xc9adbaqaaeGacaGaaiaabeqaaeqabiWaaaGcbaGaemyuae1aa0baaSqaaiabdIfayjabdMfazbqaaiabiEda3iabiwda1aaaaaa@3193@ value of this gene pair is 102.5, the metacorrelation coefficient of the reporters of probe set *258508_at *is 0.55. The examples presented here show that reporters that align best to the off-target *Y *have the most correlated signal with it and that the number of well aligning reporters plays an important role in the effect of cross-hybridization. For example, the *X *probe set in our second example has several reporters with highly correlated signal profiles to the target: the four reporters that have perfect sequence similarity with it, as well as two others with alignment scores of 107 and 89. The Pearson correlation coefficient of the summarized expression pattern of this probe set pair is high in both expression datasets (0.70 and 0.89). In the first example five reporters show relatively high signal correlation to the off-target gene. The correlation of the summarized probe set values are 0.63 and 0.62. Different to these two, the probe set pair in our third example has a comparable QXY75
 MathType@MTEF@5@5@+=feaafiart1ev1aaatCvAUfKttLearuWrP9MDH5MBPbIqV92AaeXatLxBI9gBaebbnrfifHhDYfgasaacPC6xNi=xH8viVGI8Gi=hEeeu0xXdbba9frFj0xb9qqpG0dXdb9aspeI8k8fiI+fsY=rqGqVepae9pg0db9vqaiVgFr0xfr=xfr=xc9adbaqaaeGacaGaaiaabeqaaeqabiWaaaGcbaGaemyuae1aa0baaSqaaiabdIfayjabdMfazbqaaiabiEda3iabiwda1aaaaaa@3193@ value but only two reporters show high signal correlation to gene *Y*. The correlation coefficient of this pair's expression pattern is much lower (0.30 and 0.16).

### Effect of individual reporters on probe set summaries

**Figure 5:**
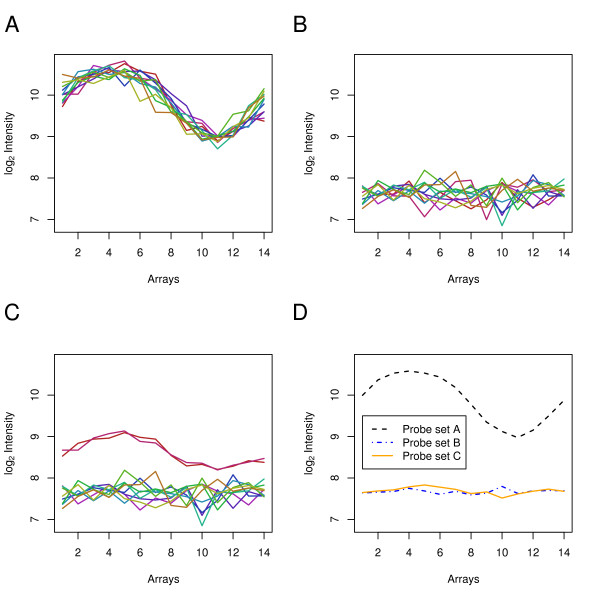
**Effect of individual reporters on probe set summaries**. A) The expression profiles of the reporters of a probe set *A *that binds the transcript of a target gene with a sinusoidal expression pattern. Each reporter is drawn in a different color. B) The expression profiles of eleven reporters of a probe set *B *that show random signals without any underlying trend. Each reporter is drawn in a different color. C) Nine of the reporters of a probe set *C *have identical expression values as nine of those of probe set *B*. Two other reporters of this probe set cross-hybridize with the transcript of gene *A *and thus have a expression pattern that is highly similar to the reporters of probe set *A*. The expression values of these two reporters are colored red. The other nine have the same colors as the corresponding reporters of probe set *B *in Figure 5B. D) The expression patterns of these three probe sets after summarization with *median polish *[15,39,40].

It may come as a surprise that a few reporters out of 11 can affect the summarized expression profile of a probe set to the extent that their inclusion coerces it to resemble that of another gene. To better understand how this can happen, consider the following simulated data example. Assume that a gene *A *has a sinusoidal expression pattern over the course of 14 time points in an experiment. Figure 5A shows the signal profiles of the 11 reporters of this gene's probe set, with data simulated using an established error model for microarray data [38]. The 11 reporters of a probe set *B *in Figure 5B show random signals without any underlying trend. Nine of the reporters of probe set *C *have identical signals as nine reporters of probe set *B*, while the remaining two reporters cross-hybridize with the transcript of gene *A *(Figure 5C). The summarized expression values obtained by applying the median polish method [39] are shown in Figure 5D. Interestingly, the Pearson correlation between probe set *A *and *B *is -0.07, while the correlation between *A *and *C *is 0.73. What is the explanation for this? The RMA method [15,39,40] exploits the fact that sensitivity to target abundance is strongly reporter-dependent and repeatable across arrays. RMA fits a model that explains the measured intensities as the product of a reporter effect and the target abundance. It estimates the model parameters, and hence the target abundance, with an outlier resistant method called *median polish*. These estimates can, however, be susceptible to subtle changes in the data, especially when the data from the reporters disagree, like here in our simulation [41].

We also explored other summarization methods. With dChip [16,42] for example, the effect of the two contaminating reporters is even stronger: the correlation between *A *and *B *is 0.30, while it is 0.95 between *A *and *C*. The statistical model that dChip uses is similar to the one of RMA, however, there are differences in the variance assumptions and the robust estimation algorithm. Affymetrix' MAS 5 software uses an algorithm called one-step Tukey's Biweight [43]. This algorithm appears to be less influenced by the two off-target reporters: the correlation between probe set *A *and *B *is -0.22, while it is -0.19 between *A *and *C*.

## Conclusion

Microarrays are an important source of functional data. Many inferential tools are based on the presence or absence of correlation in the expression profiles of genes, for example when inferring co-expression networks [1-5], in the study of the evolution of gene duplicates or families [11,12] and in the inference of gene regulatory networks [6] or Bayesian networks [7-10].

Different research groups have pinpointed the critical concern of cross-hybridization for microarray analysis [18-30]. Dai et al. [21] and Zhang et al. [20] highlighted problematic reporter annotation and underscored the importance of up-to-date reporter mappings. Zhang et al. [20] showed that about 10% of the reporters on widely-used arrays are non-specific in that they target multiple transcripts and approximately another 10% miss their target. Okoniewski and Miller [30] constructed a network of different levels of interactions between reporters and transcripts, as some reporters are able to bind more than one transcript, and some transcripts can bind more than one reporter. In this network they were able to identify several hub probe sets that show a higher absolute expression signal of reporters targeted by multiple transcripts than those that target a unique transcript because they combine the signals of many available transcripts. Moreover, their analysis revealed that probe sets whose reporters have multiple matches also show higher expression correlation with each other. Wu et al. [23] described a linear relationship between spiked-in concentrations and the measured signals of reporters that were not designed to target these particular transcripts.

We described a positive relationship between the correlation of microarray gene expression profiles and the off-target sensitivity of microarray probe sets, as estimated by sequence alignment of microarray reporters to off-target genes. Probe sets that contain reporters that align well to off-target genes show correlated intensity values to these other genes (Figure 2A and 2C).

In many cases, this positive relationship is likely not due to functional relatedness of the genes, but to a cross-hybridization artifact. Three lines of argument support this statement: first, the positive trend is present even between gene pairs that do not share longer stretches of sequence similarity and where the reporter to off-target alignment is only based on short near-matches (Figures 2A versus 2B and 2C versus 2D). Second, this effect can be observed within probe sets (Figures 3 and 4). Third, omitting reporters liable to cross-hybridization results in decreased artifactual correlation coefficients between probe sets (Figures 2B versus 2D).

Different summarization methods perform differently when dealing with cross-hybridizing reporters: methods that do majority weighting of reporters, such as RMA [15], can become unstable when there are two disagreeing groups of reporters that are close to balancing each other and when small changes can lead to a flip of the majority from one side to the other. Examples for this are shown in Figures 4 and by simulation. Simpler methods that are based on averages or trimmed averages, such as MAS [43], appear to be less affected by this problem, however, such methods suffer from the serious disadvantage of an overall smaller sensitivity [41,44]. The latter thus cannot be regarded as a solution for the cross-hybridization problem.

The standard probe set definition, as made available by the manufacturer of the array, Affymetrix, was compared to a custom-made one. In Affymetrix' definition, a probe set is a fixed set of reporters that is annotated to those genes to which a particular number of its reporters align perfectly. Probe sets can contain up to a certain number of reporters with perfect sequence identity to an off-target gene. In the custom-made CDF, a probe set is a set of reporters that align perfectly and uniquely to one gene's transcript. The use of more stringent probe set mapping and annotation results in decreased artifactual correlation coefficients. This will improve the quality of downstream analysis results. Our probe set definition is highly similar to the one used by Dai et al. [21]. Our results support and provide further evidence for the beneficial effect of probe set reorganization they and others [20] reported.

In conclusion, off-target sensitivity is a factor that should be taken into account when doing correlation analysis from microarray data. High-quality assignment of reporters to target genes is essential for inferring genuine biological expression correlations. The correlation coefficient calculated between alignment strength and expression correlation coefficients, the metacorrelation coefficient, is a novel method to identify instances of unreliable reporter behavior.

## Methods

All analyses, except for the alignments, were done with development versions of R 2.6.0 [45] and Bioconductor 2.1 [46] packages. An R package, *XhybCasneuf*, containing a reproducible compendium of the datasets and scripts used for this study, is made available and is distributed through Bioconductor [47].

### Two Chip Description Files

This analysis was carried out on the GeneChip *Arabidopsis *ATH1 genome array of Affymetrix [48]. For Affymetrix' annotation of the probe sets, a file was downloaded from the Affymetrix website [49] on August 12th, 2007. Affymetrix requires a 100% match of reporter's sequence to a consensus gene sequence and assigns a probe set to a particular locus if nine or more of the reporters in the probe set match it. We filtered out probe sets which Affymetrix assigned to multiple transcripts in addition to those that are assigned to a gene model that is not present in the TAIR6 [35] sequence database.

For the custom-made chip description file, *Exonerate *[50] was used to map reporters onto the genome and transcripts. The target sequences were the predicted transcripts from the TAIR6 release, including mitochondrial and chloroplast-encoded genes. These sequences include UTRs but not introns. The fasta file was downloaded from TAIR [51] on August 10th, 2007. We selected reporters that have perfect sequence identity with a single target gene's transcript. Reporters that hybridize with one mismatch to another gene's transcript are filtered out. We also filtered out reverse complementary matching reporters, and reporters that hybridize multiple times on the genomic sequence. The latter was done in order to remove reporters that match unannotated sequences. We included probe sets in this study if they consisted of at least eight reporters which resulted in 19,937 unique probe sets. The custom-made CDF is also available and distributed through Bioconductor ([47], *tinesath1cdf*).

### Reporter-to-transcript alignments

Reporter-to-transcript alignment scores were obtained with *Needle*, a global Needleman-Wunsch [34] alignment tool [33]. The analysis was carried out on the TAIR6 release of the *A*rabidopsis genome. The target sequences were the predicted transcripts, including mitochondrial and chloroplast-encoded genes and include UTRs but not introns. These cDNA sequences were downloaded from TAIR [52] on November 9, 2006. We ran the alignment analysis twice, with a gap penalty of -10 and -50. The same conclusions were reached but our findings were stronger when this penalty was set to -50. This means that higher correlation coefficients can be observed for reporter-to-transcript alignments without gaps.

### Microarray data

The microarray data we used were generated within the framework of the AtGenExpress project [36]. The first set is a subset of the development dataset [53] and contains the expression data of genes in 14 plant tissues. The second contains expression data of plants under nine different abiotic stress conditions [54], measured over six different time points. Both datasets were normalized using RMA [15,39,40], summarized using a median polish algorithm and averaged over replicates.

### Identification of gene pairs with long stretches of sequence similarity

To identify possibly functionally related gene pairs, we carried out a within-genome, all-against-all BLASTP [37]. Gene pairs with an E-value smaller than 10^-10 ^in at least one direction were set aside during different parts of this study.

### Metacorrelation

The metacorrelation was obtained as follows: for a probe set pair *X *and *Y*, the Pearson correlation coefficient was calculated between the alignment scores of *X*'s reporters to the transcript sequence of *Y *and the (Pearson) signal correlation coefficient of these reporters to the expression pattern of *Y*. We used the non-parametric measure for this metacorrelation because of the limited number of datapoints for each observation.

## Authors' contributions

TC designed the study, analyzed data, and wrote the paper. YVdP wrote the paper. WH designed the study, supervised the project, and wrote the paper. All authors read and approved the final manuscript.

## Supplementary Material

Additional file 1 Off-target scores of Custom-made versus Affymetrix CDF. Barplot of the off-target sensitivity scores QXY75
 MathType@MTEF@5@5@+=feaafiart1ev1aaatCvAUfKttLearuWrP9MDH5MBPbIqV92AaeXatLxBI9gBaebbnrfifHhDYfgasaacPC6xNi=xH8viVGI8Gi=hEeeu0xXdbba9frFj0xb9qqpG0dXdb9aspeI8k8fiI+fsY=rqGqVepae9pg0db9vqaiVgFr0xfr=xfr=xc9adbaqaaeGacaGaaiaabeqaaeqabiWaaaGcbaGaemyuae1aa0baaSqaaiabdIfayjabdMfazbqaaiabiEda3iabiwda1aaaaaa@3193@ of all probe set pairs in the Affymetrix (in pink) and the custom-made CDF (in light blue). This figure only shows pairs with an QXY75
 MathType@MTEF@5@5@+=feaafiart1ev1aaatCvAUfKttLearuWrP9MDH5MBPbIqV92AaeXatLxBI9gBaebbnrfifHhDYfgasaacPC6xNi=xH8viVGI8Gi=hEeeu0xXdbba9frFj0xb9qqpG0dXdb9aspeI8k8fiI+fsY=rqGqVepae9pg0db9vqaiVgFr0xfr=xfr=xc9adbaqaaeGacaGaaiaabeqaaeqabiWaaaGcbaGaemyuae1aa0baaSqaaiabdIfayjabdMfazbqaaiabiEda3iabiwda1aaaaaa@3193@ ≥ 80. Click here for file
