# Supplementary figures and images for: In situ analysis of cross-hybridisation on microarrays and the inference of expression correlation
Source: BMC Bioinformatics. 2007 Nov 26;8:461. doi: 10.1186/1471-2105-8-461 (PMC2213692; doi:10.1186/1471-2105-8-461)

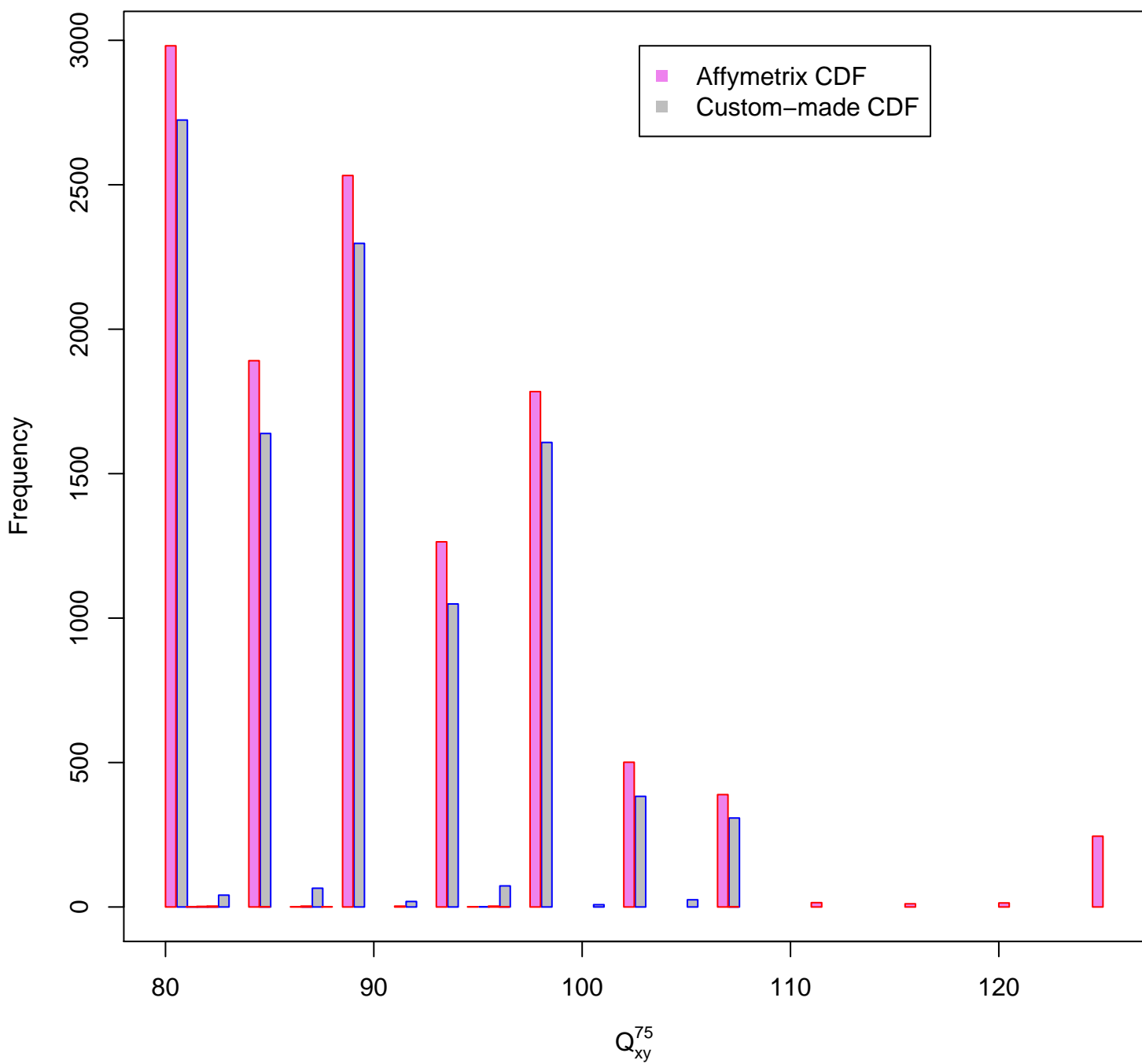

Supplement: Additional file 1 — Off-target scores of Custom-made versus Affymetrix CDF. Barplot of the off-target sensitivity scores QXY75 MathType@MTEF@5@5@+=feaafiart1ev1aaatCvAUfKttLearuWrP9MDH5MBPbIqV92AaeXatLxBI9gBaebbnrfifHhDYfgasaacPC6xNi=xH8viVGI8Gi=hEeeu0xXdbba9frFj0xb9qqpG0dXdb9aspeI8k8fiI+fsY=rqGqVepae9pg0db9vqaiVgFr0xfr=xfr=xc9adbaqaaeGacaGaaiaabeqaaeqabiWaaaGcbaGaemyuae1aa0baaSqaaiabdIfayjabdMfazbqaaiabiEda3iabiwda1aaaaaa@3193@ of all probe set pairs in the Affymetrix (in pink) and the custom-made CDF (in light blue). This figure only shows pairs with an QXY75 MathType@MTEF@5@5@+=feaafiart1ev1aaatCvAUfKttLearuWrP9MDH5MBPbIqV92AaeXatLxBI9gBaebbnrfifHhDYfgasaacPC6xNi=xH8viVGI8Gi=hEeeu0xXdbba9frFj0xb9qqpG0dXdb9aspeI8k8fiI+fsY=rqGqVepae9pg0db9vqaiVgFr0xfr=xfr=xc9adbaqaaeGacaGaaiaabeqaaeqabiWaaaGcbaGaemyuae1aa0baaSqaaiabdIfayjabdMfazbqaaiabiEda3iabiwda1aaaaaa@3193@ ≥ 80. [file 1471-2105-8-461-S1.pdf]
